# Rasorian Doses of Tartar Emetic in Tetanus

**Published:** 1860-07

**Authors:** 


*Rasorian Doses of Tartar Emetic in Tetanus.*—Dr. Cornaz relates, in *L'Echo Medical Suisse* of the 1st inst., two cases of tetanus in which repeated doses of tartar emetic were given. One of the patients, aged sixty-three, whose primary lesion was mortification of a finger, recovered; the other, aged forty-one, who had had two fingers crushed, sank under the tetanic attacks. The doses of tartar emetic were, in both cases, half a grain every half-hour; these produced rather abundant alvine evacuations, but not very severe vomiting. Both patients had, however, large doses of morphine and chlorate of potash, with warm baths. When the emetic acted too powerfully on the intestinal canal, the administration of the salt was suspended for several hours.

